# Health and social care workers’ professional values: A cross-sectional study

**DOI:** 10.1177/09697330231200569

**Published:** 2023-09-30

**Authors:** Piiku Pakkanen, Arja Häggman-Laitila, Miko Pasanen, Mari Kangasniemi

**Affiliations:** 8058 University of Turku; 205537 University of Eastern Finland; 8058 University of Turku

**Keywords:** Cross-sectional study, health and social care workers, professional ethics, professional values, student

## Abstract

**Background:**

Professional values create a basis for successful collaboration and person-centred care in integrated care and services. Little is known about how different health and social care workers assess their professional values.

**Research aim:**

To describe and compare professional value orientation among different health and social care workers in Finland.

**Research design:**

A quantitative cross-sectional study.

**Participants and research context:**

We carried out an online survey of health and social care workers from 8 March to 31 May 2022, using the Finnish version of the Nurses’ Professional Values Scale-3. The data were analysed using descriptive and advanced statistics.

**Ethical considerations:**

Permission was received from all participating organizations and those who completed the survey provided informed consent.

**Results:**

A total of 1823 health and social care workers, representing seven professional groups and students, took part. The overall level of professional values among the participants was relatively high. Commitment to providing patients and clients with equal care was more important than engaging with society and professional responsibilities in the work environment. Professional values were strongest among professionals with higher educational degrees and training in professional ethics. The same was true for workers who received organizational support for ethical practice, were satisfied with their work and had shorter work experience.

**Discussion:**

Our results showed shared professional values among different health and social care workers and students. These results are meaningful for integrated care and services. At the same time, a clear need for strengthening engagement with society and professional responsibilities for developing work environments were identified.

**Conclusions:**

Health and social care workers and students need training in professional ethics and organizational support for ethical practice and work satisfaction to maintain their professional values at different stages of their career.

## Introduction

Professional values create the foundation for health and social care workers’ daily practice. They are based on mutual goals and the values of individual professions. However, there is an increasing focus on the shared values of different professions in order to ensure that patients and clients benefit from ethical person-centred care and services.^1,2^ Professions are expected to produce integrated, joint and accessible services.^3,4^

Health and social care have increasingly used integrated strategies to respond to care and service needs, due to changes in demographics,^5^ staff shortages^6^ and collaboration in various environments and service systems.^7,8^ In addition, collaboration is required to provide safe and open practices as patients develop greater knowledge about their rights to make decisions about their own care.^1,9^

Professional values indicate the starting point for work and what it should be based on. They also show what drives a profession and what is important for its members. These values provide a framework for the duties, rights and responsibilities that individuals and professionals, as a group, have to comply with to meet their care and service obligations.^10^ Professional values are described in professional ethics^11,12^ or codes of conduct,^13^ to guide professionals’ ethical decision-making. Professional ethics is a form of self-regulation and members of the profession commit to the values voluntary.^10,14,15^ Health and social care workers display their professional value orientation by the importance they place on professional values.^15^

Health and social care professionals need to understand the legal requirements, professional ethics and values when they perform their own work and operate in integrated work environments. There are a large number of health and social care professionals and that is why providing care and services according to professional values is extremely important, as this ensures the best care for patients and clients. Despite this, little is known about how different health and social care workers assess the professional values that guide their work.

## Background

Professional values have been investigated in individual health and social care professionals, such as registered nurses^16,17^ and nursing students.^18,19^ But they have not been studied to the same extent among different health and social care workers.^20^ In previous studies, the importance of professional values among nurses and nursing students referred to the degree of the workers’ commitment to care, how active they were in society and how they conducted their responsibilities in practice.^15,16,18^ Values have been linked to workers’ personal characteristics, such as age and gender, educational and work-related issues and personal values.^21^ When they were actioned, professional values related to good patient care, patients’ satisfaction with the care they received and professionals’ job satisfaction.^22^

In social care, professional values have highlighted respect for human dignity, integrity and the right to justice and equality.^12^ In physiotherapy, the meaning of equality, confidentiality and caring were the most important professional values that were addressed.^23^ Professionals providing health and social care and services shared values, such as human dignity, equality and justice, and promoted the health, wellbeing and safety of patients and clients.^1,2^ Professional values were important in relation to the development of professional identity,^24^ clinical competence and implementing evidence-based practice^25^ and ethical decision-making.^26^

Successful mutual collaboration requires awareness of, and respect for, the professional values of other professions.^27^ However, integrating the different professions that provide patient care and services has been challenging, because they have had little knowledge and understanding of each other’s professional values.^28^ Thus, collaboration between different professions has exposed ethical issues.^20,29,30^ Ethical issues in care have been connected to different caring roles among various health and social care workers^20^ and the realization of patients’ autonomy and rights during the care process.^20,29^ Also, the professionals’ commitment to care,^20^ and the relative strength of professions,^20,30^ have been related to ethical issues. This hindered providing good quality person-centred care.

### Aim

The aim of this study was to describe and compare professional value orientations among different health and social care workers. This knowledge can be used to enhance collaboration to secure good quality care and services for clients and patients. It can also be used to develop training programmes on professional ethics and to support professionals in their daily work.

The research questions in this study were:

(1) What were the professional values among different health and social care workers?
(2) How were professional values associated with the personal characteristics of health and social care workers?

## Methods

### Design

Our quantitative, cross-sectional online survey and data collection were carried out in Finland, from 8 March to 31 May 2022. The data were analysed by descriptive and advanced statistical methods and reported according to the Strengthening the Reporting of Observational Studies in Epidemiology guidelines.^31^

### Sample and recruitment of participants

Convenience sampling was used to ensure the representativeness of different health and social care workers.^32^ The potential participants that we targeted had a degree or were degree students, were working in health and social care, and were members of professional trade unions during the data collection period. The fact that at least 10 participants responded to each survey item indicated that there were an adequate number of participants.^33^ We sought as many participants as possible, so that we could cover the various health and social care professions.

The participants were recruited with the help of 13 Finnish trade unions and professional associations. Eleven of these represented health professionals: bioanalysts, dental assistants, dental hygienists, dental technician, laboratorians, midwives, nurses, occupational therapists, optometrists, pedicurists, physiotherapists, podiatrists, public health nurses and radiographers. One represented social care professionals: social workers with Bachelor degrees, care and assistive workers, geriatric nurses, social advisors, and social care workers. One represented both health and social care professionals: care and assistive workers, childcare and youth workers, nurses and practical nurses. The contact person at each trade union shared a letter about the study with their members using emails, monthly newsletters and/or private social media groups. The letter described the aim of the study, invited people to take part and provided a link to online questionnaire. It was sent to 111,085 health and social care workers.

### Data collection

The study used the Finnish version of the Nurses Professional Values Scale-3^15^ (F-NPVS-3) to collect data and measure professional values among different health and social care workers. The scale was developed based on previous literature^15^ and ethical codes.^14^ It comprises 28 items with a five-point Likert-scale from one for not important to five for most important. The items are grouped into three factors. Caring reflects the professional’s fundamental commitment to providing equal care for patients and clients as individuals, families, groups, communities or populations. Activism reflects the professional’s duties in activities that advance their profession and its responsibilities to the public. Professionalism relates to responsibility for the work environment and practice. The total scale can range from 28 to 140. Caring and activism can both range from 10 to 50 and professionalism from 8 to 40. Higher scores reflect the higher importance that participants place on professional values and indicate what Weis and Schank^15^ described as stronger professional value orientation. The authors^15^ reported Cronbach’s alpha of 0.940 for the total scale and other studies have reported alpha values of 0.910^34^ and −0.967.^16^

A five-phase cross-cultural translation process^35^ was used to adapt the scale to the Finnish context and to ensure the translated scale matched the original. The first phase was for a qualified translator to translate the original scale from English to Finnish. The second phase was synthesis, where the translator and research group reached a consensus on the concepts related to the phenomenon. The third phase was the backward translation to English by another qualified translator. The fourth phase was shared decisions by the expert research group on the translation, culture and language of the scale. The final phase was a pilot study^35^ to evaluate the clarity of the scale instructions, items, response options and background variables. The 20 participants who were recruited were qualified health and social care professionals. As a result of this, minor corrections were made to expressions by the expert group to improve the substantive clarity of the items.^35^ The pilot study data were not included in this study.

### Data analysis

The R program, version 4.0.2^36^ (R Foundation, Vienna, Austria) was used to create descriptive and inferential statistics for the data. Frequencies and percentages are used to present the categorical data and means and standard deviations are used for the continuous descriptive data. Current work experience in health and social care was categorized as under 2 years and then in 10-year increments. General work experience was divided into 5-year periods. Participants were divided into seven professional groups, according to their degrees, and students (Table 1). Spearman’s correlation, the Kruskal–Wallis’s H test and Mann–Whitney U-test were used to explore associations between background variables and sums for total scales and factors. The Dunn test with Bonferroni correction was used for pairwise comparisons. A linear regression model was used to clarify how independent background variables explained variations in professional values among health and social care workers. Bootstrapped confidence intervals were calculated if model residuals were not normally distributed. Cross-tabulation was conducted to clarify possible reciprocal connections between independent background variables of professional groups and participating in ethics training. Due to the low number of participants, professional groups that focused on early childhood education and youth guidance and students, were excluded from the regression analysis. Cronbach’s alpha was calculated to assess internal consistency. We excluded incomplete data that exceeded 30%. The significance level was *p* < 0.05, with a confidence level of 95%.

**Table 1.** Seven professional groups divided according to participants’ degrees and students.

Professional group	Degree, *n*
Care assistants	Practical nurses, 275
	Dental assistants, 107
	Orderly, 51
	Nursing assistant, 30
	Developmental disability nurses, 28
	Instrument technician, 18
	Pedicurists, 6
	Other care and assistive workers, 57
Nurses	Public health nurses, 202
	Nurses, 117
	Midwives, 14
Diagnostic care nurses	Bioanalysts, 98
	Radiographers, 77
	Laboratorians, 53
	Optometrists, 7
Rehabilitation workers	Physiotherapists, 206
	Podiatrists, 11
	Rehabilitation instructors, 10
	Occupational therapists, 4
	Prosthetist-orthotist, 1
Social workers	Bachelors of social work, 147
	Social workers, 51
	Social advisors, 6
Oral health nurses	Dental hygienists, 99
	Dental technician, 1
Childcare and youth workers	Child nurses, 28
	Youth workers, 21
	Children’s instructors, 14
	Childminders, 13
Students	Students, 55

### Validity, reliability and rigour

The Kaiser–Meyer–Olkin test (0.963) and Bartlett’s sphericity test (*p* < 0.001) were used to verify the suitability of the data for factor analysis. Exploratory factor analysis (EFA) was used, the principal axis factoring as extraction method, with Varimax rotation.^15^ Eigenvalues higher than one were judged to be acceptable and the required communality for items was set at >0.30. The EFA for the F-NPVS-3 produced three factors, as in the original scale model by the developers.^15^ These three factors explained 50.108% of the total variance: caring (40.676%), activism (6.412%) and professionalism (3.019%). Factor structure was also confirmed with scree plots. Factor loadings ranged between 0.436 and −0.747. Communalities in items ranged between 0.332 and −0.631. Nine of the items simultaneously loaded more than one factor due to possible conceptual redundancy in the provisions of the American Nurses Association codes.^14^ This was also reported by the developers.^15^ In the EFA for the F-NPVS-3, three items were primarily loaded differently to the developers, but they simultaneously loaded to the original factors. Item 21 loaded to the activism instead of caring factor, item 27 loaded to the caring instead of activism factor and item 28 loaded to the caring instead of professionalism factor. After discussions in the research group, we decided to remove these items back to factors as in the original scale,^15^ due to the theoretical structure of the F-NPVS-3. In addition to the EFA, we conducted inter-item correlation (0.533–0.745) and item-to-total analyses (0.507–0.717) to ensure the construct validity of the F-NPVS-3. Cronbach’s alpha for entire scale was 0.929 (*n* = 1.823) and it was 0.878, 0.912, and 0.865, respectively, for caring, activism and professionalism.

### Ethical considerations

The principles of research ethics were followed throughout the study process.^37^ According to Finnish legislation, studies need ethical approval.^38^ However, this type of study did not need this, as the study participants were legally competent adults.^39^ All the organizations that took part evaluated the study and provided permission for their members and students to take part. The participants were informed about the aim and voluntary, anonymous and confidential nature of the study. They were told that they had the right to withdraw participation during any phase of the study.^40^ Informed consent was obtained electronically in the first instance and was further confirmed by completing the survey.

## Results

### Personal characteristics

The 13 Finnish trade unions and professional associations approached 111,085 health and social care workers and students and 2609 (2.35%) took part in the survey. We excluded 786 incomplete responses, and 1823 participants from seven professional groups and students were included in the statistical analyses. Most were female (91%) of Finnish origin (99%) and their mean age was 47.52 ± 11.46 years (Table 2). More than half had a degree from a university of applied sciences (59%). There were 29 different degrees representing the following professional groups and their students: care assistants (31%), nurses (18%), diagnostic care nurses (13%), rehabilitation workers (13%), social workers (11%), oral health nurses (6%), childcare and youth guidance (4%) and students (3%) (Table 2). Their work experience in health and social care varied and ranged from less than 1 year to 52 years (Table 3).

**Table 2.** Personal characteristics of the study participants.

Background variable	*n*^1^ (%)
Age (years) (*n* = 1819)	
Mean age	47.52 ± 11.46 (range 18–77)
<35	334 (18%)
36–45	369 (20%)
46–55	573 (32%)
>55	543 (30%)
Gender (*n* = 1823)	
Female	1654 (91%)
Male	135 (7%)
Other/don’t want to say	34 (2%)
Ethnicity (*n* = 1823)	
Finnish	1806 (99%)
Not Finnish	17 (1%)
Education level (*n* = 1822)	
Secondary level	554 (30%)
University of applied sciences	1078 (59%)
Other university	139 (8%)
Student	45 (3%)
Professional group (*n* = 1823)	
Care assistants	572 (31%)
Nurses	333 (18%)
Nurses in diagnostic care	239 (13%)
Rehabilitation workers	232 (13%)
Social workers	204 (11%)
Nurses in oral health	100 (6%)
Childcare and youth workers	76 (4%)
Students	55 (3%)
Professional ethics’ training in last 5 years (*n* = 1701)	
Yes	599 (35%)
No	1102 (65%)

^1^*n* in this table varies among background variables according to number of responses.

**Table 3.** Work-related characteristics of the study participants.

Background variable	*n*^1^ (%)
Work experience (years)	
In current work (*n* = 1768)	Mean 12.64 ± 11.34 (range <1–51)
<2	351 (20%)
3–10	588 (33%)
11–20	406 (23%)
>20	423 (24%)
In health and social care (*n* = 1708)	Mean 18.79 ± 12.24 (range <1–52)
<5	290 (17%)
6–15	474 (28%)
16–25	405 (24%)
>25	539 (32%)
Professional sector (*n* = 1774)	
Health care	1132 (64%)
Social care	519 (29%)
Other	123 (7%)
Employment sector (*n* = 1793)	
Public sector	1284 (72%)
Private sector (private services, third sector)	485 (27%)
Other	24 (1%)
Support for ethical practice by superior (*n* = 1807)	
Totally agree	524 (29%)
Somewhat agree	753 (42%)
Somewhat disagree	255 (14%)
Disagree	129 (7%)
Don’t know	146 (8%)
Support for ethical practice by organization (*n* = 1810)	
Totally agree	422 (23%)
Somewhat agree	864 (48%)
Somewhat disagree	315 (17%)
Disagree	101 (6%)
Don’t know	108 (6%)
Satisfied with work (*n* = 1814)	
Totally agree	496 (27%)
Somewhat agree	958 (53%)
Somewhat disagree	272 (15%)
Disagree	60 (3%)
Don’t know	28 (2%)
Form of employment (*n* = 1814)	
Permanent employee	1530 (85%)
Locum	189 (10%)
Student	59 (3%)
Don’t know	31 (2%)

^1^*n* in this table varies among background variables according to number of responses.

### Professional values among different health and social care workers

The total mean score for the importance of professional values among Finnish health and social care workers and students was high (117.06 ± 14.52, range 54–140). So were the individual scores that made up that total: caring (45.14 ± 4.80), activism (38.41 ± 6.84) and professionalism (34.04 ± 4.45) (Table 4). In general, nurses reported that professional values were most important. When professions were compared, the scores on professional values from care assistants, childcare and youth workers and nurses, in general, were statistically significantly higher than those from diagnostic care nurses. In addition, nurses generally felt that professional values were more important than social workers (Table 5).

**Table 4.** F-NPVS-3 total scale results among all health and social care workers.

Item	Mean (SD)	*n* ^1^
Caring		
Chronbach’s alpha 0.878 (*n* = 1748)		
Practice guided by principles of fidelity and respect for person	4.77 (0.48)	1807
Protect health and safety of the patient/public	4.72 (0.55)	1821
Safeguard patient’s right to confidentiality and privacy	4.69 (0.58)	1808
Respect the inherent dignity, values and human rights of individuals	4.65 (0.61)	1819
Protect moral and legal rights of patients	4.60 (0.64)	1815
Confront practitioners with questionable or inappropriate practice	4.58 (0.67)	1815
Accept responsibility and accountability for own practice	4.50 (0.68)	1816
Provide care without bias or prejudice to patients and populations	4.41 (0.76)	1813
Act as a patient advocate	4.15 (0.92)	1815
Protect rights of participants in research	4.05 (0.94)	1812
Activism		
Chronbach’s alpha 0.912 (*n* = 1729)		
Engage in consultation/collaboration to provide optimal care	4.40 (0.73)	1809
Promote mutual peer support and collegial interactions to ensure quality care and professional satisfaction	4.23 (0.80)	1811
Actively promote health of populations	4.13 (0.88)	1811
Assume responsibility for meeting health needs of diverse populations	4.09 (0.87)	1816
Take action to influence legislators and other policy makers to improve health care	3.73 (0.98)	1811
Participate in nursing research and/or implement research findings appropriate to practice	3.65 (0.93)	1807
Recognize the role of professional nursing associations in shaping health policy	3.62 (0.97)	1817
Participate in professional efforts to advance global health	3.62 (0.99)	1815
Establish collaborative partnerships to reduce health care disparities	3.56 (0.98)	1814
Advance the profession through active involvement in health-related activities	3.40 (1.01)	1813
Professionalism		
Chronbach’s alpha 0.865 (*n* = 1780)		
Recognize professional boundaries	4.60 (0.64)	1820
Assume responsibility for personal well-being	4.57 (0.64)	1815
Establish standards as a guide for practice	4.28 (0.77)	1818
Seek additional education to update knowledge and skills to maintain	4.26 (0.80)	1819
Engage in ongoing self-evaluation	4.19 (0.80)	1819
Participate in peer review	4.18 (0.78)	1814
Promote and maintain standards where planned learning activities for students take place	4.04 (0.84)	1818
Initiate actions to improve environments of practice	3.94 (0.87)	1813

^1^*n* in this table varies among variables according to number of responses.

**Table 5.** Associations between professional values, professional groups and education.

	Total scale F-NPVS-3		Caring		Activism		Professionalism	
Background variable	*p*-value	Mean score	*p*-value	Mean score	*p*-value	Mean score	*p*-value	Mean score
Professional group	<0.001		<0.01		<0.001		<0.001	
Care assistants		117.49		45.15		39.04		33.95
Nurses		119.12		45.68		39.03		34.85
Nurses in diagnostic care		113.86		43.98		36.82		33.45
Rehabilitation workers		116.84		45		38.1		34.23
Social workers		115.16		45.56		37.37		33.09
Nurses in oral health		118.12		45.05		38.83		34.66
Childcare and youth workers		118.95		45.97		39.47		33.95
Students		118.89		45.52		38.58		34.73
Educational level	0.637		0.614		0.137		0.891	
Secondary level		117.26		45.09		38.95		33.83
University of applied science		116.94		45.11		38.15		34.17
University		116.95		45.61		38.22		33.85
Students		118.42		45.44		38.8		34.22
Training in professional ethics	<0.05		0.107		<0.05		<0.05	
Yes		118.36		45.52		39.01		34.45
No		116.56		45.03		38.15		33.88

The most important factor for all the professions was caring, as this reflected values of fidelity, respect and personal and public safety and safeguarding the patient’s rights (Table 4). We found statistically significant differences when we compared the professions, as nurses and childcare and youth workers felt that caring was more important than nurses in diagnostic care (Table 5).

The least important value for all the professions was activism, which reflected their duties and responsibilities in relation to influencing society, reducing healthcare disparities and advancing global health (Table 4). When we compared the professions, we found that activism was more important to care assistants and nurses than nurses working in diagnostic care (Table 5). The differences were statistically significant.

The most important values in professionalism for all the professions were recognizing professional boundaries and an individual’s responsibility for their personal well-being. Taking action to improve the environment in which they practiced was less important (Table 4). When we compared the professions, we found that nurses reported that professionalism was more important than nurses in diagnostic care and social workers. These differences were statistically significant (Table 5).

### Personal characteristics associated with professional values

Participants who had undergone training in professional ethics during the last 5 years reported that activism, professionalism and professional values were more important, in general, than those who had not undergone such training (Table 6).

**Table 6.** Mean scores and differences in professional values among professional groups.

	Total scale F-NPVS-3		Caring		Activism		Professionalism	
Professional groups	*p*-value	Mean scores	*p*-value	Mean scores	*p*-value	Mean scores	*p*-value	Mean scores
Care assistants and Nurses in diagnostic care	<0.05	117.49 vs. 113.86			<0.01	39.04 vs. 36.82		
Nurses in diagnostic care and Nurses	<0.001	113.86 vs. 119.12	<0.01	43.98 vs. 45.68	<0.01	36.82 vs. 39.03	<0.01	33.45 vs. 34.85
Nurses and Social workers	<0.05	119.12 vs. 115.16					<0.001	34.85 vs. 33.09
Nurses in diagnostic care and Childcare and youth workers	<0.05	113.86 vs. 118.95	<0.05	43.978 vs. 45.97				

Participants with less than 5 years of general work experience said that activism and professional values were, in general, more important than participants with more than 15 years of work experience. Subjects working in social care services reported that caring was more important than those who worked in healthcare services. The participants’ workplace was associated with activism, but differences were not found in pairwise comparisons (Table 7).

**Table 7.** Associations between professional values and work-related characteristics.

	Total scale			Caring			Activism			Professionalism		
Background variable	*p*-value	*r*	Mean score	*p*-value	*r*	Mean score	*p*-value	*r*	Mean score	*p*-value	*r*	Mean score
Work experience												
In current work (years)ˆ	<0.05	0.05		0.064	−0.05		0.173	−0.03		0.109	−0.04	
In general (years)	<0.05			0.073			<0.05			0.079		
<5			119.52			45.76			39.42			34.7
6–15			116.97			44.92			38.61			33.79
16–25			116.68			45.4			37.79			33.98
>25			116.69			45.12			38.28			34.11
Work sector	0.866			0.716			0.312			0.832		
Public			117.13			45.06			38.54			34.07
Private			116.75			45.36			37.92			33.92
Other			117.56			45.4			38.74			34.09
Workplace	0.192			<0.001			<0.05			0.663		
Social care			117.61			45.87			38.67			33.85
Health care			116.75			44.89			38.18			34.11
Other			118.29			45.03			39.71			34.32
Form of employment	0.554			0.991			0.49			0.484		
Permanent employee			116.84			45.14			38.31			33.97
Substitute			118.1			45.02			38.96			34.37
Student			118.73			45.21			38.88			34.76
Other			117.45			44.71			38.96			34.34
Support for ethical practice												
By superior	<0.001	0.09		<0.01	0.07		<0.01	0.08		*<*0.0001	0.10	
By organization	<0.0001	0.10		<0.0001	0.10		<0.001	0.08		<0.001	0.08	
Satisfaction with work	<0.01	0.07		<0.01	0.07		<0.05	0.05		<0.001	0.09	

*r* = Spearman’s correlation coefficient. ˆDifferences were not found in pairwise comparisons.

Methods: Kruskal–Wallis, Spearman.

Caring, activism, professionalism and professional values, in general, were more important to participants who reported receiving support for ethical practice from their superior or organization than to those who got no such support or were not satisfied with their work (Table 7). The same was true for those who were satisfied with their work.

Regression analysis suggested that there were differences between professional groups and how they reported the importance of caring*,* activism, professionalism and professional values in general. Individual participants who were educated to university level saw caring as more important (β 1.09) than participants who were educated to secondary level (Table 8). Those who worked in social care services reported that commitment to caring was more important than participants who worked in healthcare services (β 1.03). The more organizational support for ethical practice the participants received, the higher the scores were for the general importance of professional values (β 1.3), and caring (β 0.5). The more participants were satisfied with their work, the higher scores were for the general importance of professional values (β 1.5), caring (β 0.7) and professionalism (β 0.4) (Table 8).

**Table 8.** Linear regression analysis of variation in professional values and related characteristics among health and social care workers.

	Total			Caring			Activism			Professionalism		
Background variable	β	95.0% CI	*p*	β	95.0% CI ˄	*p*	β	95.0% CI ˄	*p*	β	95.0% CI	*p*
Intercept	116.24			43.75			38.00			33.63		
Age	−0.05	−0.16, 0.06		−0.02	−0.06, 0.03		−0.02	−0.08, 0.04		−0.02	−0.06, 0.01	
Professional group												
Care assistants	Ref			Ref			Ref			Ref		
Nurses	−1.25	−4.77, 2.28		−0.32	−1.47, 0.95		−0.89	−2.78, 0.82		0.02	−1.06, 1.11	
Nurses in diagnostic care	−5.37	−9.15, −1.60	^**^	−1.62	−2.96, −0.25	^*^	−2.55	−4.48, −0.67	^**^	−1.14	−2.30, 0.03	
Rehabilitation workers	−2.92	−6.70, 0.85		−0.86	−2.08, 0.51		−1.27	−3.09, 0.54		−0.42	−1.58, 0.75	
Social workers	−7.14	−10.96, −3.31	^***^	−1.54	−2.88, −0.17	^*^	−3.75	−5.63, −1.65	^***^	−1.84	−3.03, −0.66	^**^
Nurses in oral health	−0.82	−5.33, 3.69		−0.66	−2.36, 1.00		−0.45	−2.80, 2.01		0.02	−1.38, 1.42	
Education level												
Applied sciences	ref			ref			ref			ref		
Secondary level	−1.96	−5.25, 1.34		−0.64	−1.81, 0.67		−0.43	−2.17, 1.32		−0.49	−1.51, 0.53	
University	2.28	−0.66, 5.23		1.09	0.17, 2.03	^*^	1.43	0.02, 2.80		0.50	−0.43, 1.42	
Training in professional ethics during past 5 years												
Yes	ref			ref			ref			ref		
No	−1.11	−2.72, 0.50		−0.28	−0.81, 0.25		−0.58	−1.35, 0.25		−0.36	−0.86, 0.14	
Work experience												
General	−0.01	−0.11, 0.08		0.01	−0.03, 0.05		−0.02	−0.06, 0.04		0.01	−0.02, 0.04	
Work place												
Healthcare	Ref			Ref			Ref			Ref		
Social care	2.00	−0.22, 4.22		1.03	0.29, 1.81	^**^	1.08	−0.15, 2.19		0.28	−0.41, 0.97	
Other	0.09	−3.64, 3.83		−0.5	−1.85, 0.80		0.01	−2.05, 2.07		−0.04	−1.20, 1.13	
Working sector												
Public	ref			ref			ref			ref		
Private	−0.85	−2.73, 1.02		0.31	−0.33, 0.90		−0.93	−1.81, −0.01		−0.39	−0.98, 0.19	
Other	0.69	−3.50, 4.88		0.04	−1.31, 1.14		0.03	−2.17, 2.05		0.05	−1.27, 1.36	
Support for ethical practice by												
Superior	−0.42	−1.57, 0.74		−0.23	−0.61, 0.16		0.04	−0.56, 0.59		0.08	−0.28, 0.44	
Organization	1.34	0.04, 2.64	^*^	0.51	0.04, 0.97	^*^	0.49	−0.12, 1.11		0.23	−0.18, 0.63	
Satisfaction with work												
	1.46	0.27, 2.65	^*^	0.67	0.21, 1.13	^**^	0.52	−0.07, 1.14		0.42	0.05, 0.79	^*^

β: estimate of regression coefficient. Positive β reflects higher score in professional values, and negative Beta reflects lower score in professional values.

CI: 95% confidence interval for β (lower bound–upper bound).

^˄^: Bootstrapped percentage confidence intervals because the residuals were not normally distributed.

*p < 0.05, **p < 0.01, ***p < 0.001.

## Discussion

This study provides new knowledge on the professional values of health and social care workers. We were unable to find any other studies on this or any associated personal characteristics. Previous studies have focused on the professional values of individual professions.^12,15,16,23^ Our findings showed that health and social care workers evaluated their general professional values as high. They showed strong commitment to caring, which Weis and Schank^15^ said reflected providing patients with equal care and services. This provides a meaningful basis for integrating care and services. Societal activism and professionalism in the work environment were less important professional values. These findings indicate a clear need to clarify how common values can further improve professional collaboration and the good quality care provided to clients and patients.

### Shared professional values on care and services for patients and clients

Based on our findings, all health and social care workers recognized the importance of caring, considering ethical values, such as patient’s rights and fidelity, respect and safety. Our results indicate a strong commitment to providing equal care and services. These are values that have been described in connection with the professional ethics of health and social care workers^11,12^ and multi-professional ethics.^41^ The results are also in line with previous studies that showed that the highest importance was placed on caring values.^15,21^ This is particularly meaningful in patient groups where professionals typically work together, for example, in older people’s care. Respecting the dignity, worldview views and human rights of each other is essential in this area. As a result, a crucial part of professional value orientation is the shared understanding of professional values in care, as this will result in integrated person-centred care.^18^

Health and social care workers agreed that less important professional values were engaging with wider society and responsibilities related to the work environment and nursing practice. These results were in line with previous studies.^21,25^ However, it is important to consider societal engagement in future,^17,25,34^ because it would influence the quality of local and global care and services. Traditionally, changing the culture of practice and learning how to influence society has not actually played much of a role in the content of health and social care and services. One study reported that participatory governance supported and empowered workers’ engagement in decision-making and developing practice.^42^ We need to clarify societal engagement, responsibility for the work environment and promoting practice as a professional value, and incorporate these into ethics and leadership education.

Health and social care workers considered their own well-being as highly important values in professionalism, as well as recognizing their professional boundaries. In line with previous studies, the new generation of health and social care workers are particularly likely to take their own well-being into account.^6,43^ Traditionally, health and social care workers have placed patients above everything, based on their calling to provide care and services. They were expected to forget their own well-being and yield to professional boundaries.^43,44^ However, the COVID-19 pandemic is one factor that has seriously challenged health and social care workers with regard to their own well-being.^45,46^ Taking care of themselves and setting professional boundaries has helped workers to cope with new situations at work.^47^ In addition, leadership styles have affected the well-being of workers.^48^ Therefore, although the well-being of patients and clients is still the primary concern, health and social care workers must also focus on their own well-being and their ability to stay in their job. These need to be openly discussed, without any guilt, in both organizations and society.

### Importance of education and organizational support to professional values

The results of this study, that training in professional ethics strengthened perceptions of professional values, agreed with previous studies.^17,49^ However, the result that professional values were highest after graduating, but weakened when work experience increased, was at odds with other studies.^21,49,50^ These studies found that educational courses, scientific meetings and self-directed learning in ethics may have affected the perceptions of nurses and health care workers with regard to their professional values. Our results clearly challenge education providers to strengthen training in professional ethics among health and social care workers, as this will strengthen their shared values at different stages of their careers. Shared values will enable them to meet the requirements of integrated services and provide high-quality person-centred care in collaboration with other health and social care workers.

In this study, organizational support for ethical practice and work satisfaction was associated with professional values for all health and social care workers. According to Poikkeus et al.,^51^ organizational support was related to nurses’ possibilities to practice in accordance with their professional values. However, they also reported that nurses and nurse leaders reported low knowledge of values in other professions. The authors^51^ also indicated that professionals received more support to work in accordance with their professional values in larger organizations than small care units.^51^ In line with a previous study,^22^ high levels of professional values increased work satisfaction among nurses as well as patients’ satisfaction in the care they received. Health and social care organizations of all sizes need strategies to guide and support their workforce to maintain strong professional value orientation and ethical values during daily practice need to be highlighted. In an organizational context, ethical committees^51^ have addressed how to enable professionals to reflect on shared values, as well considering and learning about the mutual basis of values.

### Limitations

The limitations of this study concerned the recruitment, participation rate and representativeness of the participants. More than 100,000 members were contacted by their trade unions and professional associations about the study, but only 2609 enrolled in the study and less than 2000 fully completed the questionnaire. Reminders were sent, but they did not result in any significant increase. Probability sampling might have increased the response rate, but we used convenience sampling because we wanted to make sure that everyone who was approached had the chance to take part. It is worth noting that there was a national nursing strike during the study as well as the COVID-19 pandemic. It is also possible that only those who were interested in the study phenomenon took part. The NPVS scale was originally designed^15^ and used to measure the development and sustainability of professional values in nurses^15^ and nursing students.^15,34^ Compatibility between the F-NPVS-3 and the ethical codes and shared ethical principles of Finnish health and social care workers were assessed. No discrepancies were observed between the original and target cultures or professions. The number of female participants who took part in the study was high, but this was in line with the Finnish target population. In addition, the study data were skewed when we compared the representation of different health and social care workers and the relative strengths between their professions in the field.^52^ However, the data were comprehensive, covered seven different professional groups, plus students, and satisfactorily represented different health and social care workers and students in Finnish health and social care.^52^ This improved the generalizability of the results.

## Conclusions

This study produced highly positive findings on professional values among different health and social care workers, with regard to providing integrated care. The findings indicate a commitment to providing patients and clients with equal care and services. However, they also highlight a clear need for further training in professional ethics and values, especially in relation to the ability to engage society and the professional skills needed to develop work environments and practice. This study shows that it is important for health and social care organizations to provide their staff with support, so that they can practice their profession in an ethical way. They also need to be provided with professional training at different stages of their careers, so that they can support shared professional values and thus implement person-centred care in collaboration with other professionals. Future studies should focus on professional values among different health and social care workers in relation to changes in society and services. In addition, similar professional value orientation does not explain ethical issues in daily practice. That is why it is meaningful to explore collaboration among different health and social care workers and how their shared values could help the common goal of providing high-quality care to clients and patients. In future, we need to examine how leadership could support professional values among different health and social care workers and evaluate the types of organizational support that are most effective. It is also necessary to examine how patients and their close relatives perceive professional values in care and services.

## References

[1] Kallio H Häggman-Laitila A Saarnio R , et al. Working towards integrated client-oriented care and services: a qualitative study of the perceptions of Finnish health and social care professionals. Int J Care Coord 2022; 25: 46–52.

[2] Karam M Brault I Van Durme T , et al. Comparing interprofessional and interorganizational collaboration in healthcare: a systematic review of the qualitative research. Int J Nurs Stud 2018; 79: 70–83. 29202313 10.1016/j.ijnurstu.2017.11.002

[3] Baxter S Johnson M Chambers D , et al. The effects of integrated care: a systematic review of UK and international evidence. BMC Health Serv Res 2018; 18: 350. DOI: 10.1186/s12913-018-3161-3. 29747651 PMC5946491

[4] Nummela O Juujärvi S Sinervo T . Competence needs of integrated care in the transition of health care and social services in Finland. Int J Care Coord 2019; 22: 36–45.

[5] World Health Organization [WHO] . Global action plan on the public health response to dementia 2017–2025. Geneva: World Health Organization. License: CC BY-NC-SA 3.0 IGO, https://www.who.int/publications/i/item/9789241513487 (2017, accessed 27 February 2023).

[6] Drennan VM Ross F . Global nurse shortages – the facts, the impact and action for change. Br Med Bull 2019; 130: 25–37. 31086957 10.1093/bmb/ldz014

[7] Auschra C . Barriers to the integration of care in inter-organisational settings: a literature review. Int J Integrated Care 2018; 18: 1–14. 10.5334/ijic.3068 PMC5887071 29632455

[8] World Health Organization [WHO] . European office. Integrated care models: an overview. Working document. Health services delivery programme, division of health systems and public health, https://www.euro.who.int/__data/assets/pdf_file/0005/322475/Integrated-care-models-overview.pdf (2016, accessed 27 February 2023).

[9] Lawless MT Marshall A Mittinty MM , et al. What does integrated care mean from an older person’s perspective? A scoping review. BMJ Open 2020; 10: e035157. DOI: 10.1136/bmjopen-2019-035157. PMC7044957 31974092

[10] Kangasniemi M Pakkanen P Korhonen A . Professional ethics in nursing: an integrative review. J Adv Nurs 2015; 71: 1744–1757. 25598048 10.1111/jan.12619

[11] International Council of Nurses [ICN] . The ICN code of ethics for nurses. Revised 2021, https://www.icn.ch/system/files/2021-10/ICN_Code-of-Ethics_EN_Web_0.pdf (2021, accessed 27 February 2023).

[12] International Federation of Social Workers [IFSW] . Global social work statement of ethical principles, https://www.ifsw.org/global-social-work-statement-of-ethical-principles/ (2018, accessed 27 February 2023).

[13] American Physical Therapy Association [APTA] . APTA guide for conduct of the physical therapist assistant, https://www.apta.org/contentassets/7cbd42e5a7e94740a07bf790b9b79fc6/apta-guide-for-conduct-pta.pdf (2010, accessed 27 February 2023).

[14] American Nurses Association [ANA] . Code of ethics for nurses with interpretive statements. Maryland: Silver Spring, https://www.nursingworld.org/practice-policy/nursing-excellence/ethics/code-of-ethics-for-nurses/ (2015, accessed 27 February 2023).

[15] Weis D Schank MJ . Development and psychometric evaluation of the nursing professional values scale - 3. J Nurs Meas 2017; 25: 400–410. 29268825 10.1891/1061-3749.25.3.400

[16] Asiandi A Erlina M Lin Y-H , et al. Psychometric evaluation of the nurses professional value scale-3: Indonesian version. Int J Environ Res Publ Health 2021; 18(16): 8810. DOI: 10.3390/ijerph18168810. PMC8391371 34444553

[17] Poreddi V Narayanan A Thankachan A , et al. Professional and ethical values in nursing practice: an Indian perspective. Invest Educ Enfermería 2021; 39(2): e12. DOI: 10.17533/udea.iee.v39n2e12. PMC8253520 34214289

[18] Venables H Wells Y Fetherstonhaugh D , et al. Factors associated with nursing students’ attitudes toward older people: a scoping review. Genrontol Geriatr Educ 2023; 44: 131–150. 10.1080/02701960.2021.2012466 34927567

[19] Poorchangizi B Borhani F Abbaszadeh A , et al. Professional values of nurses and nursing students: a comparative study. BMC Med Educ 2019; 19(1): 438. DOI: 10.1186/s12909-019-1878-2. 31775723 PMC6882014

[20] Pakkanen P Häggman-Laitila A Kangasniemi M . Ethical issues identified in nurses’ interprofessional collaboration in clinical practice: a meta-synthesis. J Interprof Care 2022; 36: 725–734. 34120556 10.1080/13561820.2021.1892612

[21] Gassas R Salem O . Factors affecting nurses’ professional values: a comprehensive integrative review. Nurse Educ Today 2022; 118: 105515. DOI: 10.1016/j.nedt.2022.105515. 36030580

[22] Kaya A Boz I . The development of the professional values model in nursing. Nurs Ethics 2019; 26: 914–923. 28929939 10.1177/0969733017730685

[23] Arnal-Gómez A Muñoz-Gómez E Espí-López GV , et al. Professional values and perception of knowledge regarding professional ethics in physical therapy students: a STROBE compliant cross-sectional study. Medicine 2022; 101(35): e30181. DOI: 10.1097/MD.0000000000030181. 36107566 PMC9439820

[24] Fitzgerald A . Professional identity: a concept analysis. Nurs Forum 2020; 55: 447–472. 32249453 10.1111/nuf.12450

[25] Skela-Savic B Hvalic-Touzery S Pesjak K . Professional values and competencies as explanatory factors for the use of evidence-based practice in nursing. J Adv Nurs 2017; 73: 1910–1923. 28205259 10.1111/jan.13280

[26] Chen Q Su X Liu S , et al. The relationship between moral sensitivity and professional values and ethical decision-making in nursing students. Nurse Educ Today 2021; 105: 105056. DOI: 10.1016/j.nedt.2021.105056. 34265538

[27] Goldman J Kitto S Reeves S . Examining the implementation of collaborative competencies in a critical care setting: key challenges for enacting competency-based education. J Interprof Care 2018; 32: 407–415. 29161170 10.1080/13561820.2017.1401987

[28] Kangasniemi M Karki S Voutilainen A , et al. The value that social workers’ competencies add to health care: an integrative review. Health Soc Care Community 2021; 30: 403–414. 33704859 10.1111/hsc.13266

[29] Podgorica N Flatscher-Thöni M Deufert D , et al. A systematic review of ethical and legal issues in elder care. Nurs Ethics 2021; 28: 895–910. 32468910 10.1177/0969733020921488

[30] Sturm A Edwards I Fryer CE , et al. (Almost) 50 shades of an ethical situation – international physiotherapists’ experiences of everyday ethics: a qualitative analysis. Physiother Theory Pract 2023; 39: 351–368. 34983285 10.1080/09593985.2021.2015812

[31] Von Elm E Altman DG Egger M , et al. The strengthening the reporting of observational studies in epidemiology (STROBE) statement: guidelines for reporting observational studies. Int J Surg 2007; 12(12): 1495–1499. DOI: 10.1016/j.ijsu.2014.07.013. 25046131

[32] Grove SK Burns N Gray JR . The practice of nursing research. Appraisal, synthesis, and generation of evidence. 7th ed. St. Louis, Missouri: Elsevier Saunders, 2013, pp. 363–364.

[33] Sousa VD Rojjanasrirat W . Translation, adaptation and validation of instruments or scales for use in cross-cultural health care research: a clear and user-friendly guideline. J Eval Clin Pract 2011; 17: 268–274. 20874835 10.1111/j.1365-2753.2010.01434.x

[34] Alabdulaziz H Alasmee NA Almazan JU . Psychometric analysis of the Nurses’ Professional Values Scale-3 Arabic version among student nurses. Int Nurs Rev 2022; 69: 221–228. 33899940 10.1111/inr.12677

[35] Beaton DD Bombardier C Guillemin G , et al. Guidelines for the process of cross-cultural adaptation of self-report measures. Spine 2000; 25: 3186–3191. 11124735 10.1097/00007632-200012150-00014

[36] The R Foundation . R foundation for statistical computing, https://www.r-project.org/foundation/ (accessed 27 February 2023).

[37] All European Academies . The European code of conduct for research integrity. Revised edition https://ec.europa.eu/research/participants/data/ref/h2020/other/hi/h2020-ethics_code-of-conduct_en.pdf (2017, accessed 27 February 2023).

[38] The Medical Research Act (488/1999) . Ministry of social affairs and health. Retrieved from https://www.finlex.fi/en/laki/kaannokset/1999/19990488 (1999, accessed 27 February 2023).

[39] Finnish National Board on Research Integrity TENK . The ethical principles of research with human participants and ethical review in the human sciences in Finland. Finnish national board on research integrity TENK guidelines 2019. Publications of the Finnish national board on research integrity TENK 3/2019, https://tenk.fi/sites/default/files/2021-01/Ethical_review_in_human_sciences_2020.pdf (2019, accessed 5 August 2023).

[40] EU . Regulation (EU) 2016/679, of the European Parliament and of the council of 27 April 2016 on the protection of natural persons with regard to the processing of personal data and on the free movement of such data, and repealing directive 95/46/EC (general data protection regulation). https://eur-lex.europa.eu/legal-content/EN/TXT/PDF/?uri=CELEX:32016R0679&from=EN (2016, accessed 27 February 2023).

[41] Interprofessional Education Collaborative [IPEC] . Core competencies for interprofessional collaborative practice: 2016 update. Washington, DC: Interprofessional Education Collaborative. https://ipec.memberclicks.net/assets/2016-Update.pdf (2016, accessed 27 February 2023).

[42] Kanninen T Häggman-Laitila A Tervo-Heikkinen T , et al. Nursing shared governance at hospitals – it’s Finnish future? Leader Health Serv 2019; 32: 558–568. 10.1108/LHS-10-2018-0051 PMC7324080 31612781

[43] Hult M Halminen O Mattila-Holappa P , et al. Health and work well-being associated with employment precariousness among permanent and temporary nurses: a cross-sectional survey. Nord J Nurs Res 2022; 42: 140–146.

[44] Kallio H Kangasniemi M Hult M . Registered nurses’ perceptions of having a calling to nursing: a mixed-method study. J Adv Nurs 2022; 78: 1473–1482. 35188282 10.1111/jan.15157 PMC9306482

[45] Ditwiler RE Swisher LL Hardwick DD . Professional and ethical issues in United States acute care physical therapists treating patients with COVID-19: stress, walls, and uncertainty. Phys Ther 2021; 101: 1–10. 10.1093/ptj/pzab122 PMC8136052 33956143

[46] Turale S Meechamnan C Kunaviktikul W . Challenging times: ethics, nursing and the COVID-19 pandemic. Nursing and health policy perspectives. Int Nurs Rev 2020; 67: 164–167. 32578249 10.1111/inr.12598 PMC7361611

[47] De Kock JH Latham HA Leslie SJ , et al. A rapid review of the impact of COVID-19 on the mental health of healthcare workers: implications for supporting psychological well-being. BMC Publ Health 2021; 21: 104. DOI: 10.1186/s12889-020-10070-3. PMC7794640 33422039

[48] Niinihuhta M Häggman-Laitila A . A systematic review of the relationships between nurse leaders’ leadership styles and nurses’ work-related well-being. Int J Nurs Pract 2022; 28: 1–22. 10.1111/ijn.13040 PMC9788052 35102648

[49] Poorchangizi B Farokhzadian J Abbaszadeh A , et al. The importance of professional values from clinical nurses’ perspective in hospitals of a medical university in Iran. BMC Med Ethics 2017; 18(1): 20. DOI: 10.1186/s12910-017-0178-9. 28249603 PMC5333397

[50] Monroe AH (2019). Nurses’ professional values: influences of experience and ethics education. J Clin Nurs 2019; 28: 2009–2019. 30706591 10.1111/jocn.14806

[51] Poikkeus T Suhonen R Katajisto J , et al. Organisational and individual support for nurses’ ethical competence: a cross-sectional survey. Nurs Ethics 2018; 25: 376–392. 27165144 10.1177/0969733016642627

[52] Statistics Finland . Health and social services personnel, https://www.stat.fi/en/statistics/sthlo (2021, accessed 27 February 2023).

